# Baby blues, premenstrual syndrome and postpartum affective disorders: intersection of risk factors and reciprocal influences

**DOI:** 10.1192/bjo.2023.612

**Published:** 2023-12-04

**Authors:** Natalia Chechko, Elena Losse, Thomas Frodl, Susanne Nehls

**Affiliations:** Department of Psychiatry, Psychotherapy and Psychosomatics, Faculty of Medicine, RWTH Aachen, Aachen, Germany; JARA-Institute Brain Structure Function Relationship (INM-10), Institute of Neuroscience and Medicine, Research Center Jülich, Jülich, Germany; and Institute of Neuroscience and Medicine, Brain & Behavior (INM-7), Research Center Jülich, Jülich, Germany; Department of Psychiatry, Psychotherapy and Psychosomatics, Faculty of Medicine, RWTH Aachen, Aachen, Germany; Department of Psychiatry, Psychotherapy and Psychosomatics, Faculty of Medicine, RWTH Aachen, Aachen, Germany; and JARA-Institute Brain Structure Function Relationship (INM-10), Institute of Neuroscience and Medicine, Research Center Jülich, Jülich, Germany; Department of Psychiatry, Psychotherapy and Psychosomatics, Faculty of Medicine, RWTH Aachen, Aachen, Germany; Department of Psychiatry and Psychotherapy, University Hospital, Otto von Guericke University Magdeburg, Magdeburg, Germany

**Keywords:** Baby blues, premenstrual syndrome, postpartum depression, risk factors, longitudinal study

## Abstract

**Background:**

The aetiology and consequences of ‘baby blues’ (lower mood following childbirth) are yet to be sufficiently investigated with respect to an individual's clinical history.

**Aims:**

The primary aim of the study was to assess the symptoms of baby blues and the relevant risk factors, their associations with clinical history and premenstrual syndrome (PMS), and their possible contribution to the early recognition of postpartum depression (PPD).

**Method:**

Beginning shortly after childbirth, 369 mothers were followed up for 12 weeks. Information related to their clinical history, PMS, depression, stress and mother–child attachment was collected. At 12 weeks, mothers were classified as non-depressed, or with either PPD or adjustment disorder.

**Results:**

A correlation was found between the severity of baby blues and PMS (*r* = 0.397, *P* < 0.001), with both conditions increasing the possibility of adjustment disorder and PPD (baby blues: OR = 6.72, 95% CI 3.69–12.25; PMS: OR = 3.29, 95% CI 2.01–5.39). Baby blues and PMS independently predicted whether a mother would develop adjustment disorder or PPD after childbirth (*χ*^2^(64) = 198.16, *P* < 0.001). Among the non-depressed participants, baby blues were found to be associated with primiparity (*P* = 0.012), family psychiatric history (*P* = 0.001), PMS (*P* < 0.001) and childhood trauma (*P* = 0.017).

**Conclusions:**

Baby blues are linked to a number of risk factors and a history of PMS, with both conditions adding to the risk of PPD. The neuroendocrine effects on mood need be understood in the context of individual risk factors. The assessment of both baby blues and PMS symptoms within the first postpartum days may contribute to an early identification of PPD.

Sex hormones and glucocorticoids rise to unprecedented levels during pregnancy, before plunging dramatically at parturition.^1^ Coinciding with the hormonal deficiency in the postpartum phase, a high number of women experience changes in mood commonly referred to as the ‘baby blues’, the symptoms of which include mood swings after childbirth, physical discomfort, fatigue, anxiety, irritability and uncertainty about the maternal role.^2^ Although the vast majority of cases involving baby blues resolve within approximately 2 weeks, in some women the mood disturbance may persist beyond the postpartum period, indicating that baby blues may be an early indicator of depression. Affecting approximately 17% of the world's population of postpartum women,^3^ and 8% of them in Europe,^4^ postpartum depression (PPD) occurs in direct relation (during pregnancy or within 4 weeks postpartum) to pregnancy (DMS-5^5^). Another likely event linked to childbirth is postpartum adjustment disorder, which affects approximately 13% of mothers^6^ and is frequently related to an identifiable stressor. That the symptom severity of adjustment disorder does not meet the criteria for depression^5^ at any time distinguishes the condition from PPD. Although neither adjustment disorder nor baby blues have the debilitating effects of clinical depression, both should be regarded as important differential diagnoses of PPD.^6,7^

Previous results suggest that baby blues have functional correlates in brain areas related to the development of depression.^8,9^ Therefore, the identification of women at a higher risk of PPD while they experience the symptoms of baby blues may contribute to an early recognition of the condition. However, given that baby blues are a common event following childbirth, affecting up to 50–75% of new mothers,^10,11^ assessing these symptoms alone may not be enough to allow the prediction of PPD. Additional factors may need to be taken into account. It has been suggested, for instance, that conditions such as premenstrual syndrome (PMS) may be triggered by hormonal fluctuations. Among women of childbearing age, 18% experience at least one mood symptom in the luteal phase and 2–10% are afflicted by severe PMS,^5^ the most common symptoms of which (similar to baby blues) are irritability and depressed mood.^12^ Studies that have hypothesised an association between PMS and PPD (e.g.,^13,14^) show that all three conditions (baby blues, PMS and PPD) may be experienced more strongly by those with an increased susceptibility to negative mood shifts during hormonal transition.

## Aims of the study

The main focus of the present study was to assess the symptoms of baby blues in the first few weeks following childbirth, and the relevant risk factors, their associations with the clinical history, particularly PMS, as well as their impact on PPD over a 12-week period. More than 80% of studies^3^ in the field utilise various cut-off thresholds on the self-reported Edinburgh Postnatal Depression Scale (EPDS)^15^ as a diagnostic tool for PPD and to estimate its prevalence.^3,16^ However, studies using the EPDS as a diagnostic tool report a global PPD prevalence of approximately 17%, whereas those that use the DSM criteria for diagnosis report a global prevalence of 10%,^3^ suggesting that self-report questionnaires lead to an overestimation of PPD prevalence. As previously shown by us, subsyndromal disorders such as adjustment disorder can also lead to transiently elevated EPDS scores.^6,7^ Therefore, in our study, the EPDS-based assessment of PPD was supplemented by a clinical interview, in addition to which, baby blues, PPD and PMS were assessed independently of one another by various means. That provided an opportunity to assess the similarities as well as the differences between these closely linked yet completely distinct conditions. Seeking to determine the predisposing and antecedent factors that contribute to baby blues, we hypothesised a relationship between the severity of baby blues and personal history, particularly in terms of PMS (hypothesis 1). Subsequently, we examined the effects of baby blues on postpartum well-being, hypothesising that the severity of baby blues would differentially affect both stress and the severity of the depressive symptoms (hypothesis 2). Finally, we hypothesised that the combination of baby blues and PMS, depending on their severity, would also differentially affect non-depressed women and the development of adjustment disorder and PPD (hypothesis 3).

## Method

### Participants

From the data pool of the Risk for Postpartum Depression (RiPoD) study (*n* = 865), the data of 369 women, who had completed both the Maternity Blues Questionnaire (MBQ)^2^ and the Premenstrual Tension Syndrome Scale (PTSS)^17^ and had completed the 12-week follow-up and had the final diagnosis, were collected. The recruitment of euthymic postpartum women (i.e. who had no depression at the time of recruitment, based on the DSM-5 criteria) was performed in the Department of Gynecology and Obstetrics at the University Hospital Aachen within 1–6 days of childbirth (for the recruitment procedure, see^6,7^). Prior to enrolment in the study, written informed consent was obtained from each participant. The authors assert that all procedures contributing to this work comply with the ethical standards of the relevant national and institutional committees on human experimentation and with the Helsinki Declaration of 1975, as revised in 2008. All procedures involving human participants were approved by the Institutional Review Board of the Medical Faculty, RWTH Aachen University (EK208/15).

### Questionnaires

At 1–6 days postpartum, clinical anamnestic screenings (demographic information, information about the pregnancy, individual and family psychiatric history) were carried out. Current depressive symptoms after childbirth were assessed using the EPDS,^15^ a self-report instrument with 10 items on a four-point scale (from 0 to 3), with higher sum scores indicating more depressive symptoms. General stressful and traumatic events were obtained through the Stressful Life Events Questionnaire (SLESQ),^18^ which includes possible encounters with 11 events. For the present analyses, only the ‘yes’ responses, i.e. the number of stressful life events, were counted. Childhood experiences (until the age of 18) of physical, emotional and sexual abuse and physical and emotional neglect were assessed with the Childhood Trauma Questionnaire (CTQ),^19^ which contains 28 questions requiring responses on a five-point response scale ranging from ‘being never true’ to ‘being very often true’. In addition, to help assess the perceived quality of parenting by one's own parents up to the age of 16 years, participants completed the Parental Bonding Instrument (PBI).^20^ The PBI includes 12 ‘care’ versus ‘rejection’ items and 13 ‘overprotection’ versus ‘autonomy’ items regarding one's own mother and father, answered on a four-point Likert scale (very unlikely to very likely).

One week postpartum, participants indicated symptoms of baby blues in the first postpartum week by means of the Maternal Blues Questionnaire (MBQ),^2^ a self-rating scale with 28 items and response options of ‘yes’ or ‘no.’ The method proposed by Kennerley & Gath was used to calculate the cut-off point: this uses the mean peak score of each sample, i.e. the 50th percentile, for moderate blues. The threshold for severe blues was the 90th percentile of the score distribution in the sample, as suggested by Glangeaud-Freudenthal et al^1^ and Takács et al.^22,23^ At 3, 6, 9 and 12 weeks postpartum, remote assessments sent via an email link helped assess the preceding 3 weeks by means of the EPDS, the Maternal Postnatal Attachment Scale (MPAS)^24^ and the Perceived Stress Scale (PSS).^25^ The MPAS is a 19-item self-report measure in which mothers report their feelings towards their infant and themselves as parents, each item being scored from 1 to 5, with lower sum scores meaning lower attachment and *vice versa*. The PSS is a self-report measure with 10 items on a five-point response scale, scored from 1 to 5, with higher sum scores indicating more stress.

After 12 weeks, the 36-item Premenstrual Tension Syndrome Scale (PTSS)^17^ was used to identify those with no PMS symptoms or mild ones (0–4), moderate symptoms (5–13) and severe symptoms (>14) in the week before menstruation, in addition to the total PMS score (total number of symptoms). Finally, based on DSM-5 criteria, an experienced psychiatrist or psychologist assigned participants with depressive mood either to the PPD group or the adjustment disorder group and those without depressive symptoms to the non-depressed group.

### Missing data

In the selected sample, 76.42% of participants (*n* = 282) had a complete data-set. The missing data were prominent mostly on the PBI subscale pertaining to paternal care and overprotection (11.7% missing, *n* = 43). For 32 of the 43 women, data were missing owing to absence of the father during childhood, early death or no contact. With respect to the remaining 35 variables, less than 5% of the data were missing. Furthermore, the missing data were relevant only to hypothesis 1. As for hypotheses 2 and 3, the nature of the selected sample involved no missing data (e.g. completed MBQ score and PMS score, completed follow-up and final diagnosis).

Given that no patterns were detected in the distribution of the missing data across the entire population, we assumed that the data were missing completely at random. The distribution of the missing data is provided in Supplementary Fig. 1, available at https://doi.org/10.1192/bjo.2023.612. Based on Little's MCAR test (χ^2^(433) = 434.40, *P* = 0.47), these data were deemed appropriate for imputation.

We used the multiple imputation method (*m* = 5) to deal with missing data. All characteristics were used as predictors to impute missing values, and the observed minimum and maximum values of the original full data-set were used as constraints. Analyses on the imputed data-sets were performed for each separately imputed data-set, pooling results over each data-set. The pooled estimates of the five imputed data-sets are reported.

### Statistical analyses

The statistical analyses were performed using SPSS 27.0 for Windows.

To determine whether the three baby blues groups (none, moderate, severe) as well as the three diagnostic groups (non-depressed, adjustment disorder, PPD) differed in terms of clinical anamnestic and demographic factors, analysis of variance (ANOVA) and Welch's *F*-tests or χ^2^-tests were performed.

Student's *t*-tests and Mann–Whitney *U*-tests were computed to compare the MBQ scores in different sample combinations (e.g. parity status, traumatic childbirth) and to compare non-depressed women with and without baby blues.

Spearman's correlation was used to determine whether the MBQ scores were associated with clinical anamnestic and demographic factors.

The interaction between the severity of baby blues and PMS (none, moderate, severe) and the postpartum diagnosis was calculated using a hierarchical three-way log-linear analysis. Coefficient values, odds ratios (ORs) and 95% confidence intervals (CIs) were used to quantify the strength of the associations.

In addition, to examine the influence of clinical anamnestic data on the relationship between MBQ scores and PTSS scores, a simple moderation analysis (model 1) was performed using PROCESS.^27^

To explore associations between MBQ scores, PTSS scores and the postpartum diagnosis, we performed a multinomial logistic regression with non-depressed women as the reference group and MBQ and PTSS scores as predictors. We developed a series of models adjusting for potential confounders and estimated the odds ratios of developing adjustment disorder or PPD. In addition to MBQ and PTSS scores, model 1 included sociodemographic variables (age, total number of children, marital status, degree of education, income). Model 2 included the model 1 covariates and pregnancy and child-related variables (parity, complications at birth/pregnancy, weeks of gestation, birth mode, subjectively experienced trauma, weight of the infant, relocation of the infant to a special care baby unit). Model 3 included model 2 covariates and personal history and family characteristics (prior depression, family psychiatric history, stressful life events, childhood trauma). We report unadjusted and adjusted odds ratios and 95% confidence intervals for these comparisons.

The reported *P*-values were corrected for multiple comparisons using the Benjamini–Hochberg false-discovery rate correction, when appropriate. The statistical significance for all tests was set at *P* < 0.05.

## Results

In the postpartum group (*n* = 369), 293 women (79.4%) were born in Germany, 10 (2.7%) in other West European countries (Belgium, France, The Netherlands), 2 (0.5%) in Spain, 1 (0.3%) in Norway, 27 (7.3%) in Eastern Europe (Poland, Lithuania, Romania, Russia, Slovakia, Ukraine, Hungary), 5 (1.4%) in Balkan countries (Bosnia, Kosovo, Bulgaria, Greece), 3 (0.8%) in the Middle East (Turkey, Lebanon, Iran, Iraq), 8 (2.2%) in Central, East and Southeast Asia (China, Kazakhstan, Kirgizstan, Tajikistan, Indonesia), 4 (1.1%) in Arab countries (Egypt, Morocco), 1 (0.3%) in West Africa (Liberia/Ghana), 2 (0.5%) in North America (USA, Canada) and 3 (0.3%) in South America (Venezuela, Colombia); 3 (0.8%) reported a mixed origin (Germany/Netherlands, Germany/Algeria, Germany/Poland) and 7 (1.9%) did not provide information regarding their country of origin. Sample characteristics of the blues severity groups can be found in Table 1.

**Table 1:** Sample characteristics for the postpartum women, divided into women without baby blues, with moderate baby blues and with severe baby blues

	No blues		Moderate blues		Severe blues		*P*
	Mean (s.d.)	*n* (%)	Mean (s.d.)	*n* (%)	Mean (s.d.)	*n* (%)	
Age, years	33.17 (4.49)		32.29 (4.33)		31.32 (4.75)		0.038
Married		126 (76.4)		122 (73.1)		25 (67.6)	0.534
Education							0.003
<9 years		10 (6.1)		10 (6.0)		1 (2.7)	
10–12 years		32 (19.4)		15 (9.0)		2 (5.4)	
>13 years		123 (74.5)		142 (85.0)		34 (91.9)	
Annual income, thousand €							0.043
<20		15 (9.1)		7 (4.2)		5 (13.5)	
20–50		48 (29.1)		56 (33.5)		12 (32.4)	
>50		102 (61.8)		104 (62.3)		20 (54.1)	
Primiparous		83 (50.3)		103 (61.7)		25 (67.6)	0.036
Total number of children	1.68 (0.77)		1.59 (0.84)		1.46 (0.69)		0.12
Weeks of gestation	39.28 (1.53)		38.91 (2.06)		39.01 (1.97)		0.129
Birth mode							0.211
Spontaneous		92 (55.8)		86 (51.5)		16 (43.2)	
Operative vaginal delivery		9 (5.5)		19 (11.4)		3 (8.1)	
Elective C-section		40 (24.2)		47 (28.1)		13 (35.1)	
Emergency C-section		24 (14.5)		15 (9)		5 (13.5)	
Complications during pregnancy		58 (35.2)		73 (43.7)		11 (29.7)	0.142
Complications at birth		43 (26.1)		47 (28.1)		13 (35.1)	0.537
Subjective experience of traumatic childbirth		10 (6.1)		20 (12.3)		8 (21.6)	0.012
Birthweight of child, g	3418.48 (511.96)		3417.25 (711.85)		3435.27 (755.12)		0.980
Relocation of child		41 (24.8)		42 (25.1)		17 (45.9)	0.025
Breastfeeding at childbirth		147 (89.6)		158 (94.6)		33 (89.2)	0.021
Breastfeeding at 12 weeks pp		132 (81.0)		144 (86.2)		30 (81.1)	0.407
At least one stressful live event		81 (49.1)		94 (56.3)		24 (64.9)	0.165
Number of stressful live events	0.88 (1.23)		1.23 (1.61)		1.43 (1.76)		0.009
Number of childhood traumas	31.34 (8.19)		34.83 (11.58)		35.46 (12.22)		0.002
Emotional abuse	6.83 (2.78)		8.09 (4.15)		8.19 (4.24)		0.002
Physical abuse	5.43 (1.27)		5.74 (2.01)		5.68 (1.44)		0.151
Sexual abuse	5.39 (1.53)		5.77 (2.37)		6.46 (3.41)		0.006
Emotional neglect	7.39 (3.14)		8.76 (4.05)		8.62 (4.04)		0.003
Physical neglect	6.28 (2.21)		6.48 (2.13)		6.51 (2.49)		0.406
PTSS score	4.25 (4.97)		8.23 (6.79)		10.51 (9.12)		<0.001
PMS severity							<0.001
None		115 (69.7)		65 (38.9)		12 (32.4)	
Mild		39 (23.6)		66 (39.5)		12 (32.4)	
Severe		11 (6.7)		35 (21.6)		13 (35.1)	
EPDS at childbirth	4.71 (3.81)		6.56 (4.41)		8.76 (4.76)		<0.001
EPDS 0–3 weeks pp	3.11 (2.51)		7.61 (3.12)		13.27 (5.81)		<0.001
EPDS 3–6 weeks pp	2.99 (3.30)		6.1 (4.04)		10.03 (5.81)		<0.001
EPDS 6–9 weeks pp	2.54 (3.25)		5.28 (3.89)		8.51 (5.613)		<0.001
EPDS 9–12 weeks pp	1.88 (2.59)		4.76 (4.11)		7.57 (4.86)		<0.001
MPAS 0–3 weeks pp	87.56 (4.73)		83.6 (6.07)		78.81 (8.48)		<0.001
MPAS 3–6 weeks pp	87.39 (5.43)		84.37 (6.08)		79.7 (9.25)		<0.001
MPAS 6–9 weeks pp	87.96 (4.75)		85.23 (5.92)		82.68 (7.63)		<0.001
MPAS 9–12 weeks pp	88.48 (4.52)		86.13 (5.66)		83.3 (6.78)		<0.001
PSS 0–3 weeks pp	11.25 (4.69)		16.79 (5.01)		22.46 (6.74)		<0.001
PSS 3–6 weeks pp	10.59 (5.13)		14.91 (5.39)		19.32 (7.12)		<0.001
PSS 6–9 weeks pp	9.9 (5.69)		13.46 (5.46)		16.96 (7.76)		<0.001
PSS 8–12 weeks pp	8.53 (5.00)		12.87 (5.54)		15.38 (6.34)		<0.001
Postpartum diagnosis							<0.001
Non-depressed		150 (90.9)		113 (67.7)		9 (24.3)	
Adjustment disorder		12 (7.3)		41 (24.6)		15 (40.5)	
Postpartum depression		3 (1.8)		13 (7.8)		13 (35.1)	
Prior depression (before pregnancy)		18 (10.9)		36 (21.6)		9 (24.3)	0.021
Family psychiatric history		32 (19.4)		56 (33.5)		12 (32.4)	0.011
PBI: Care from own mother	29.54 (6.99)		28.49 (7.52)		28.67 (7.63)		0.268
PBI: Overprotection by own mother	7.69 (5.80)		9.82 (7.21)		9.31 (6.47)		0.013
PBI Care from own father	27.69 (7.37)		24.85 (8.23)		23.85 (9.31)		<0.001
PBI: Overprotection by own father	7.1 (5.57)		8.84 (7.02)		9.14 (6.46)		0.017
Support at home 0–12 weeks pp	1.77 (0.80)		1.85 (0.87)		2.41 (1.32)		0.002

PTSS, Premenstrual Tension Syndrome Scale; PMS, premenstrual syndrome; EPDS, Edinburgh Postnatal Depression Scale; pp, postpartum; MPAS, Maternal Postnatal Attachment Scale; PSS, Perceived Stress Scale; PBI, Parental Bonding Instrument.

**Fig. 1:**
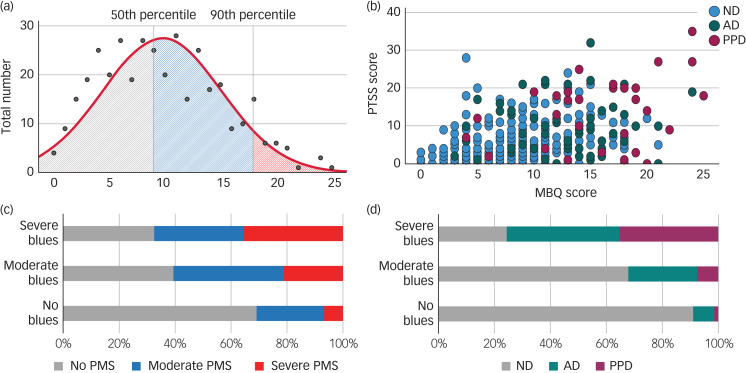
(a) Distribution of the MBQ scores across the postpartum group, with applied 50th and 90th percentile. (b) Trajectories of baby blues (MBQ score) and premenstrual syndrome (PTSS score) symptoms across the three diagnostic groups (ND, AD and PPD). Frequency distributions between group combinations with different severities of (c) baby blues and PMS, and (d) baby blues and postpartum diagnosis. ND, no depression; AD, adjustment disorder; PPD, postpartum depression; PTSS, Premenstrual Tension Syndrome Scale; MBQ, Maternity Blues Questionnaire; PMS, premenstrual syndrome.

In the total sample, the MBQ scores ranged from 0 to 25, with a mean peak score of 9.74 (s.d. = 5.36). Applying the 50th percentile results in a cut-off score of 9, indicating 165 women below this score having no baby blues (mean 4.89, s.d. = 2.24). The 90th percentile results in a cut-off score of 18, indicating 167 women with a score between 10 and 17 points (mean 12.34, s.d. = 2.41) having moderate symptoms, and 37 women with a score >18 (mean 19.68, s.d. = 2.00) having severe blues symptoms (Fig. 1(a)).

All three PMS groups differed significantly in MBQ scores (*F*(2, 368) = 35.58, *P* < 0.001), with those without PMS symptoms (*n* = 192) having the lowest blues scores (mean 7.83, s.d. = 4.96), followed by moderate PMS (*n* = 117; mean 10.86, s.d. = 4.75) and severe PMS (*n* = 60; mean 13.50, 5.13; all *P* > 0.01) (Fig. 1(c)).

Similarly, the diagnostic groups differed significantly in MBQ scores (*F*(2, 366) = 59.23, *P* < 0.001), with non-depressed women (*n* = 272) having the lowest scores (mean 8.2, s.d. = 4.59), compared with those with adjustment disorder (*n* = 68; mean 13.63, s.d. = 4.63, *P* < 0.001) and PPD (*n* = 29; mean 15.79, s.d. = 5.47, *P* < 0.001), although the latter two groups were not significantly different (*P* = 0.054) (Fig. 1(d)).

Sample characteristics for the diagnostic groups (non-depressed, adjustment disorder, PPD) and for non-depressed women divided into those with and without baby blues are provided in Supplementary Tables 1 and 2 respectively.

### MBQ scores and pregnancy-unrelated variables

In the total sample, the MBQ scores correlated negatively with age (*r* = −0.130, *P* = 0.015) and with the total number of children (*r* = −0.131, *P* = 0.015). In addition, primiparity was associated with higher MBQ scores (*r* = 0.165, *P* = 0.003). Disentangling across the postpartum diagnostic groups, associations between MBQ scores and parity status *(r* = 0.202, *P =* 0.003) or number of children (*r* = 0.198, *P* = 0.003) were found to be significant only in the non-depressed group.

In the total sample, the MBQ scores correlated positively with the number of experienced stressful events (*r* = 0.173, *P* = 0.002) and childhood traumas (*r* = 0.246, *P* < 0.001). The scores correlated negatively with the perceived care from the participants’ own parents up to the age of 16 (mother: *r* = −0.111, *P* = 0.041; father: *r* = −0.216, *P* < 0.001) as well as positively with perceived overprotection by the parents (mother: *r* = 0.139, *P* = 0.012; father: *r* = 0.158, *P* = 0.007). Again, only in the non-depressed group, the number of childhood traumas (*r* = 0.198, *P* = 0.003), particularly emotional abuse (*r* = 0.183, *P* = 0.006) and neglect (*r* = 0.202, *P* < 0.003), and maternal overprotection (*r* = 0.150, *P* = 0.03) and paternal care (*r* = −0.172, *P* = 0.015) correlated significantly with MBQ scores, whereas all other correlations as well as those within the adjustment disorder and PPD groups were non-significant.

In addition, only in the non-depressed group, a history of depression *(r* = 0.152, *P* = 0.027) and a family history of psychiatric disorders *(r* = 0.209, *P* = 0.003) were associated with MBQ scores in the first week postpartum. In women with adjustment disorder or PPD, personal and family history were not associated with higher MBQ scores.

No significant association was found with any socioeconomic variable (marital status, income, education).

### Baby blues, pregnancy and the postpartum course

No significant association was found between MBQ scores and duration of pregnancy, complications during pregnancy, mode of delivery, weight of newborn and breastfeeding. However, complications during childbirth and the subjective experience of childbirth being traumatic were significantly associated with MBQ scores *(r* = 0.113, *P* = 0.043; *r* = 0.173, *P* = 0.002). Women with severe baby blues were more likely to have had their children relocated to a special care baby unit (χ^2^(2) = 7.3, *P* = 0.026).

In the total sample, MBQ scores correlated positively with EPDS and PSS scores and negatively with MPAS scores throughout the study period (Table 2). When the postpartum groups were disentangled, again, only in the non-depressed group did all correlations remain significant.

**Table 2:** Spearman rank correlation of Maternity Blues Questionnaire scores with postpartum depression, stress and attachment variables for the total sample and the disorder subgroups (non-depressed, adjustment disorder, postpartum depression)

	Total postpartum sample	Non-depressed	Adjustment disorder	Postpartum depression
EPDS at birth	0.367**	0.227**	−0.076	0.698**
EPDS 0–3 weeks pp	0.766**	0.715**	0.617**	0.303
EPDS 3–6 weeks pp	0.564**	0.443**	0.272*	0.236
EPDS 6–9 weeks pp	0.521**	0.392**	0.101	−0.037
EPDS 9–12 weeks pp	0.524**	0.426**	0.084	0.698**
MPAS 0–3 weeks pp	−0.489**	−0.437**	−0.437**	−0.106
MPAS 3–6 weeks pp	−0.400**	−0.324**	−0.401**	−0.08
MPAS 6–9 weeks pp	−0.346**	−0.289**	−0.321**	0.072
MPAS 9–12 weeks pp	−0.335**	−0.264**	−0.226	−0.074
PSS 0–3 weeks pp	0.674**	0.615**	0.485**	0.698**
PSS 3–6 weeks pp	0.533**	0.408**	0.266*	0.245
PSS 6–9 weeks pp	0.433**	0.339**	0.006	0.131
PSS 9–12 weeks pp	0.474**	0.386**	0.079	0.159

EPDS, Edinburgh Postnatal Depression Scale; pp, postpartum; MPAS, Maternal Postnatal Attachment Scale; PSS, Perceived Stress Scale.

*P < 0.05, **P < 0.001.

### Risk profiles in non-depressed women with and without baby blues

In comparison with non-depressed women without baby blues, those with baby blues were significantly more often primiparous (χ^2^(1) = 6.32, *P* = 0.012) and showed a trend towards being younger (*t*(270) = 1.48, *P* = 0.071).

Non-depressed women with baby blues, compared with those without, were significantly more often primiparous (χ^2^(1) = 6.32, *P* = 0.012) and, in terms of trend, slightly younger (*t*(270) = 1.48, *P* = 0.071). The non-depressed women with baby blues experienced significantly more childhood emotional abuse (*P* = 0.013, *d* = 0.258), sexual abuse (*P* = 0.032, *d* = 0.236), emotional neglect (*P* = 0.012, *d* = 0.267), maternal overprotection *(P* = 0.006, *d* = 0.0326), paternal overprotection (*P* = 0.032, *d* = 0.211) and less paternal care (*P* = 0.006, *d* = 0.292). Additionally, they had significantly higher PTSS scores (*P* = 0.001, *d* = 0.617) and were more often affected by moderate and severe PMS (χ^2^(2) = 27.95, *P* = <0.001). Throughout the postpartum follow-up period, non-depressed women with baby blues had significantly higher (subclinical) EPDS scores, higher PSS scores and lower MPAS scores at all time points (Supplementary Table 2).

### Association between MBQ and PTSS scores in the total sample

To examine the relationship between MBQ and PTSS scores, a simple moderation analysis (model 1) was performed. With PTSS scores as the predictor variable and MBQ scores as the dependent variable, all pre-pregnancy history/pregnancy-unrelated variables for the participants were included individually as moderator variables. The results showed a significant interaction between PMS and SLESQ scores (*B* = −0.064, 95% CI −0.117 to −0.011, *P* = 0.017). The explained variance increased by 1.28% by adding the interaction effect (*R*^2^ change of 0.0128). The conditional effects of PMS on MBQ showed a significant positive association between PMS and MBQ across low, average and high levels of moderation (low moderation: *B* = 0.416, 95% CI 0.313–0.519, *t* = 7.949, *P* < 0.001; average moderation: *B* = 0.352, 96% CI 0.274–0.430, *t* = 8.819, *P* < 0.001; high moderation: *B* = 0.288, 95% CI 0.203–0.373, *t* = 6.650, *P* < 0.001). The Johnson–Neyman region of significance shows a significant positive effect of the moderator for SLESQ scores up to and including SLESQ = 3.989. For higher SLESQ scores, the effect of the moderator becomes non-significant.

### Linear association between the affective-symptoms severity groups

There was a significant association between the experience of baby blues (moderate and severe) and the development of a postpartum affective disorder (adjustment disorder and PPD; χ^2^(1) = 45.55, *P* < 0.001, OR =  6.72, 95% CI 3.69–12.25). Similarly, symptoms of PMS (moderate and severe) were associated with the development of a postpartum affective disorder (χ^2^(1) = 23.48, *P* > 0.001, OR = 3.29, 95% CI 2.01–5.39) and baby blues (χ^2^(1) = 37.31, *P* < 0.001, OR = 3.79, 95% CI 2.45–5.87).

A three-way log-linear analysis of the severities of the mood conditions (baby blues, PMS, AD/PPD), with a likelihood ratio of χ^2^(0) = 0, *P* = 1, indicated a significant highest-order interaction (χ^2^(8) = 18.30, *P* = 0.019). To break down this effect, three-way χ^2^-tests were computed independently for each of the variables. For each baby blues severity group, significant linear associations were between the PMS severities and the postpartum diagnoses (all *P* < 0.05; Supplementary Table 3). Similarly, for each PMS severity group, baby blues severity showed a significant linear association with the postpartum diagnoses (all *P* < 0.05; Supplementary Table 3).

However, the three-way χ^2^-test performed for the postpartum diagnostic groups resulted in a significant linear-by-linear association between the degree of baby blues and the degree of PMS only in the non-depressed group, but not in the adjustment disorder and PPD groups (Table 3).

**Table 3:** Three-way contingency tables of frequency distributions of postpartum diagnosis, baby blues severity and premenstrual syndrome (PMS) severity

	No PMS	Moderate PMS	Severe PMS	*P*
Non-depressed group				
No blues	110	34	6	<0.001
Moderate blues	49	46	18	
Severe blues	3	6	0	
Adjustment disorder group				
No blues	4	3	5	0.628
Moderate blues	13	18	10	
Severe blues	6	4	5	
Postpartum depression group				
No blues	1	2	0	0.254
Moderate blues	3	2	8	
Severe blues	3	2	8	

### Association between MBQ and PTSS scores and the development of postpartum affective disorders

In the multinomial logistic regression model with the postpartum diagnosis as dependent variable, both unadjusted and adjusted models indicated that MBQ and PTSS scores were associated with the development of adjustment disorder and PPD (χ^2^(64) = 198.16, *P* < 0.001) (Table 4). After adjustment for sociodemographic, pregnancy and child-related variables and for personal history (model 3), women in the adjustment disorder group (compared with the non-depressed group) indicated more symptoms of PMS (*B* = 0.61, s.e. = 0.28, *P* = 0.026) and of baby blues (*B* = 0.21, s.e. = 0.42, *P* < 0.001). Similarly, developing PPD was predicted by PMS (*B* = 0.12, s.e. = 0.04, *P* = 0.004) and baby blues symptoms (*B* = 0.34, s.e. = 0.73, *P* < 0.001).

**Table 4:** Multinomial logistic regression predicting postpartum diagnosis from baby blues and premenstrual syndrome symptoms^a^

	Odds ratio (95% CI)			
	Unadjusted	Model 1	Model 2	Model 3
Adjustment disorder group				
PTSS	1.11 (1.06–1.15)***	1.07 (1.02–1.12)**	1.07 (1.02–1.12)**	1.06 (1.01–1.12)*
MBQ	1.24 (1.16–1.32)***	1.23 (1.15–1.32)***	1.21 (1.13–1.30)***	1.24 (0.1.4–1.35)***
Postpartum depression group				
PTSS	1.19 (1.12–1.25)***	1.13 (1.06–1.21)***	1.14 (1.07–1.21)***	1.12 (1.04–1.22)**
MBQ	1.38 (1.26–1.52)***	1.32 (1.19–1.48)**	1.32 (1.18–1.49)***	1.41 (1.22–1.63)***

MBQ, Maternity Blues Questionnaire; PTSS, Premenstrual Tension Syndrome Scale.

a. The reference class is the non-depressed group. Model 1: adjusted for socioeconomic variables (age, total number of children, marital status, degree of education, income). Model 2: adjusted for model 1 and for pregnancy and child-related variables (parity, complications at birth/pregnancy, weeks of gestation, birth mode, subjectively experienced birth trauma, weight of the infant, relocation of the infant to a special care baby unit). Model 3: adjusted for model 2 and for personal history (prior depression, family psychiatric history, stressful life events, childhood trauma).

*P < 0.05, **P < 0.005, ***P < 0.001.

In the total sample, a highly significant correlation between the MBQ and PMS scores (*r* = 0.397, *P* < 0.001) indicated that the more severe the PMS the more severe the blues. However, disentangling this correlation within each diagnostic group resulted in a significant correlation between MBQ and PTSS scores only in the non-depressed group (*r* = 0.384, *P* < 0.001), whereas correlations for the adjustment disorder and PPD groups were non-significant.

## Discussion

In our study, 55% of the 369 participants reported having baby blues. Other studies have suggested similar rates of prevalence in European countries.^10^ The experience of baby blues was found to be associated with experiences of PMS, stressful life events, emotional trauma in childhood, traumatic childbirth as well as perceived parental overprotection and the paucity of care received as a child. Women who experienced baby blues were younger, more often primiparous, their children were more frequently relocated to a special care baby unit and they reported less support at home from their partners. In sum, women experiencing baby blues had several risk factors associated not only with the circumstances involving childbirth but also with previous experiences in life and the family of origin, confirming our first hypothesis.

### Baby blues in women experiencing PPD or postpartum adjustment disorder

In this selected cohort, more women in the PPD and adjustment disorder groups experienced baby blues (90 and 82% respectively) than in the non-depressed group (45%). In addition, 50% of women affected by baby blues in the PPD group reported to being affected severely, compared with 7% of non-depressed women and 27% of those with adjustment disorder. In PPD, the association between severity of baby blues, EPDS score and stress levels was seen only at 3 weeks postpartum. Although baby blues remain a risk factor for the development of PPD, the lack of coping strategies and stress management^28,29^ are maintaining factors in depression, which may act independently of the initial severity of the baby blues. This means that the elevated EPDS scores and stress levels seen in the follow-up of the PPD group are likely no longer related to the hormonal risk factors. Thus, partly contrary to our second hypothesis, in the PPD and adjustment disorder groups, we did not see a clear relationship between the experience of baby blues and the postpartum trajectories of EPDS scores, stress or attachment.

The experience of baby blues in women with adjustment disorder and PPD was independent of age, number of children and parity. Both groups were not only more frequently affected by baby blues but also had a much higher prevalence of traumatic events, along with personal and family psychiatric history, compared with the non-depressed group. Therefore, the lack of variance in the severity of baby blues symptoms and these factors in the PPD and adjustment disorder groups resulted in no correlation. In the entire cohort, the differences associated with baby blues were driven mostly by the non-depressed participants.

### Risk profiles of non-depressed women experiencing baby blues

The non-depressed women with baby blues were slightly younger, more frequently first-time mothers, had more often a family history of psychiatric disorder and experienced adverse parenting during their own childhood. Parity as well as childhood maltreatment can leave long-lasting marks on the endocrine system^30–32^ and influence individual sensitivity to progesterone and cortisol,^33^ making women more susceptible to hormonal fluctuations. Thus, the experience of baby blues seems to identify a more vulnerable group of women in terms of risk factors for affective reactions. That these women tend to respond affectively to hormonal fluctuations in general is reflected not only in the presence of baby blues but also in a higher percentage of non-depressed women with baby blues being affected by PMS. Additionally, levels of stress seem to have a worsening effect not only on depression, but also on the severity of PMS.^34,35^ In the presence of stress, the adrenal glands release progesterone in addition to cortisol,^36,37^ linking the functions of the hypothalamic–pituitary–gonadal axis and the adrenal axis. Therefore, it is likely that women who are less stress resistant, or suffer from more stress, react more strongly to hormonal fluctuations associated with baby blues or PMS.

### Associations between PMS, baby blues and postpartum mental health

We confirmed our final hypothesis by demonstrating strong associations between the severity of baby blues and PMS and the postpartum affective state. Although the severity of baby blues and that of PMS helped to distinguish between the non-depressed group and the two affective disorder groups, the interaction between baby blues and PMS did not. When the three diagnostic groups were considered independently of one another, the association between PMS and baby blues remained significant only in the non-depressed group. It can be postulated, therefore, that the severity of baby blues and PMS tends to decrease in non-depressed women, and the highest probability of remaining non-depressed is predicated on MBQ and PTSS scores remaining low.

Using both scales to identify women at risk of developing PPD, however, seems promising. We demonstrated a strong relationship between the presence and severity of PMS and baby blues, both of which were observed more frequently in, and were experienced in more severe forms by, women with PPD or adjustment disorder, with the PPD group being more severely affected. Given that baby blues are common,^10,11^ thus likely not specific enough to predict PPD, the combined assessment with the history and severity of PMS might prove helpful in distinguishing non-depressed women from those with PPD within 1 week of childbirth.

Baby blues, PMS and PPD can be considered as symptomatic (e.g. affective) reactions. The continuous exposure to high oestradiol, progesterone and its metabolite allopregnanolone during pregnancy has been suggested to have mood stabilising effects,^38,39^ whereas the deficiency of these hormones after parturition has been associated with baby blues and PPD.^1,40^ For instance, PMS mood symptoms increase in parallel with the rise in progesterone during the luteal phase, reaching their peak during the last 5 premenstrual days, when progesterone levels begin to decline.^41^ It has been suggested that women with PMS develop a decreased sensitivity and higher tolerance to allopregnanolone compared with their healthy counterparts^42^ and experience a greater drop in progesterone in the late luteal phase,^43^ suffering more severe withdrawal symptoms. This may be comparable to the sharp drop in progesterone following childbirth.

However, given the enormous number of different negative experiences and risk factors already prevalent in adjustment disorder and PPD, baby blues or the sensitivity to hormonal factors are unlikely to have any independent influence on the development of adjustment disorder or PPD. Thus, the neuroendocrine effects on mood must be understood in the context of individual risk factors and temporal relationships, as the presence of PMS or baby blues likely only interacts with the existing risk factors, such as stressful live events, instead of independently leading to a mental disorder.

### Limitations and strengths

In our cohort, the MBQ was administered only once, for practical reasons. Also, baby blues assessment may have been exaggerated by an already present adjustment disorder. In particular, the MBQ might not have been sensitive enough to distinguish between baby blues and depressive symptoms, especially in the first few weeks after childbirth. Furthermore, a clear distinction between these two conditions, which are both self-limiting, is not always possible. In this context, time is of crucial importance,^6^ given that baby blues typically dissipate at 2 weeks postpartum and adjustment disorder typically lasts longer.

Another limitation is the relatively small sample size in certain subgroups, e.g. the 37 women with severe baby blues or the 29 women with PPD in our total sample of 369 participants, combined with the use of several statistical methods with different requirements. Thus, it should be considered that the statistical power of our analyses may be reduced, increasing the risk of missing true effects because of insufficient data and limiting the generalisability of our results.

The strengths of the study lie in its close observation of a large and representative sample of postpartum women over a period of 12 weeks, the exclusion of prenatal depression as a confounder, and a clinical interview-based diagnosis of PPD and adjustment disorder.

### Clinical implications

Our data demonstrate that the screening of women under increased risk of developing depression may be improved by including both an assessment of baby blues severity and the history and severity of PMS. This can be done within the first few postpartum days, when the time-related criterion for PPD is not yet fulfilled (according to DSM-5,^5^ the diagnosis of PPD requires a symptom duration of at least 2 weeks). Thus, the assessment of baby blues and the history of PMS may lead to an early identification of a high-risk group for further monitoring. Close observation of symptom development by means of digital or clinical assessment of mood^6^ may aid a timely detection of the relevant symptoms.

## Supporting information

Chechko et al. supplementary material Chechko et al. supplementary material

## Data Availability

The data that support the findings of this study are available on request from the corresponding author. They are not publicly available owing to privacy or ethical restrictions.
