# Supplementary material for: Baby blues, premenstrual syndrome and postpartum affective disorders: intersection of risk factors and reciprocal influences
Source: BJPsych Open. 2023 Dec 4;10(1):e3. doi: 10.1192/bjo.2023.612 (PMC10755547; doi:10.1192/bjo.2023.612)
Supplement: Chechko et al. supplementary material [file S2056472423006129sup001.docx]

*Supplementary Information for*

**Baby blues, premenstrual syndrome and postpartum affective disorders: the intersection of risk factors and reciprocal influences**

Chechko N, Losse E, Frodl T, Stickel S

**Methods**

**
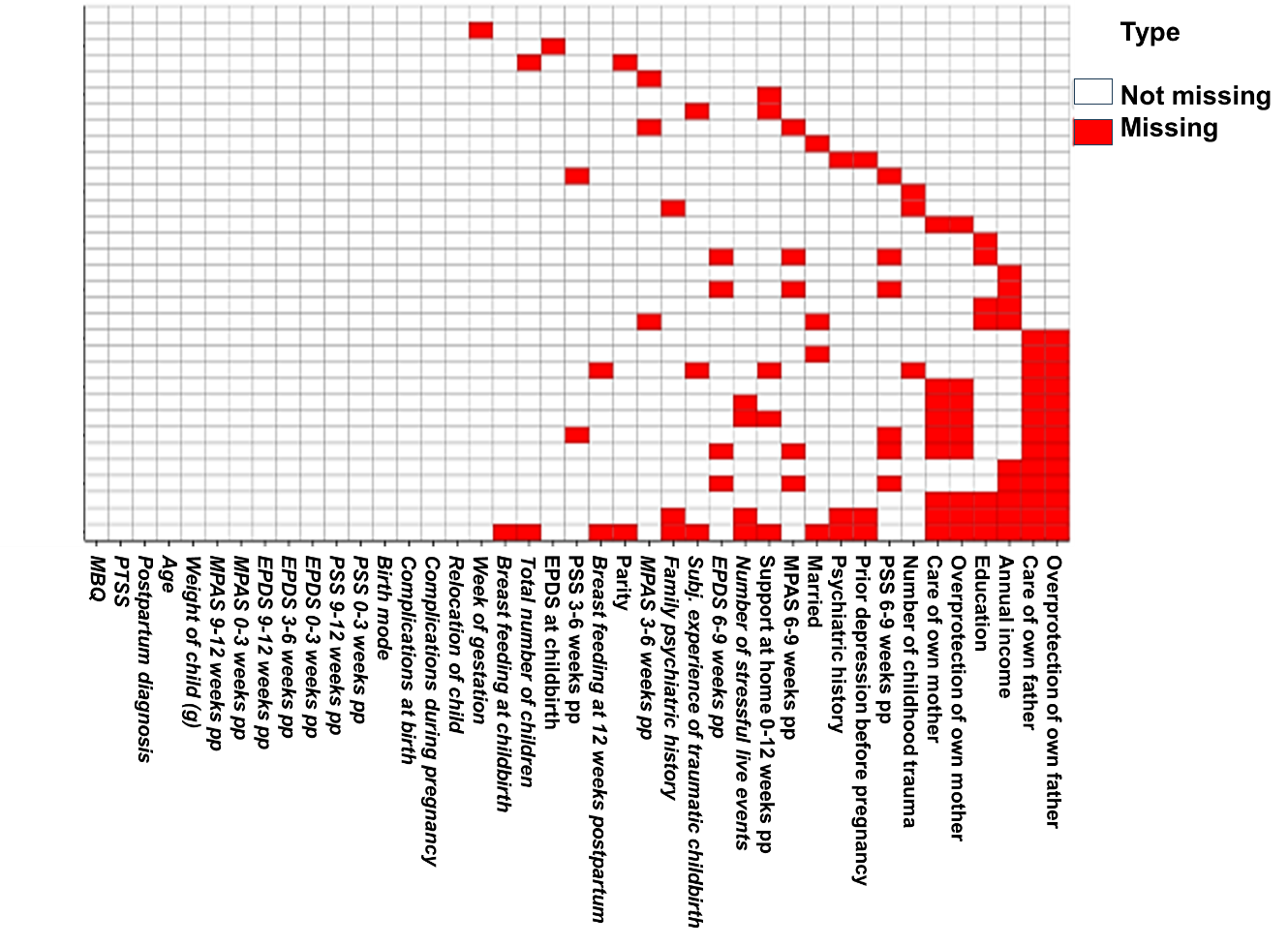
**

Figure S1. Missing data distribution map.

**Results**

Table S1. Sample characteristics (means, standard deviations, and frequencies) of postpartum women divided into non-depressed women, women with adjustment disorder and women with postpartum depression.

|  | Non-depressed |  | Adjustment disorder |  | Postpartum depression |  | p |
| --- | --- | --- | --- | --- | --- | --- | --- |
|  | ***M* (*SD*)** | ***n* (%)** | ***M* (*SD*)** | ***n* (%)** | ***M* (*SD*)** | ***n* (%)** |  |
| *Age* | 32.9 (4.26) |  | 31.85 (4.34) |  | 31.38 (6.18) |  | .121 |
| *Married* |  | 211 (77.6) |  | 44 (64.7) |  | 18 (62.1) | .05 |
| *Education* |  |  |  |  |  |  | .567 |
| *< 9 years* |  | 14 (5.1) |  | 4 (5.9) |  | 2 (6.9) |  |
| *10-12 years* |  | 39 (14.3) |  | 7 (10.3) |  | 3 (10.3) |  |
| *> 13 years* |  | 219 (80.5) |  | 57 (83.8) |  | 24 (82.8) |  |
| *Annual income* |  |  |  |  |  |  | **.026** |
| *<20k €* |  | 20 (7.3) |  | 3 (4.4) |  | 5 (17.2) |  |
| *20-50k €* |  | 78 (28.7) |  | 27 (39.7) |  | 9 (31.0) |  |
| *> 50k €* |  | 174 (64.0) |  | 38 (55.9) |  | 15 (51.7) |  |
| *Primiparous* |  | 117 (43.0) |  | 29 (42.6) |  | 13 (44.8) | .804 |
| *Total number of children* | 1.59 (0.77) |  | 1.66 (0.89) |  | 1.72 (0.84) |  | .324 |
| *Week of gestation* | 39.18 (1.66) |  | 38.77 (2.48) |  | 39.94 (1.65) |  | .174 |
| *Birth mode* |  |  |  |  |  |  | .626 |
| *spontaneous* |  | 150 (55.1) |  | 31 (45.6) |  | 13 (44.8) |  |
| *ventouse* |  | 21 (7.7) |  | 7 (10.3) |  | 3 (10.3) |  |
| *elective c-section* |  | 68 (25.0) |  | 23 (33.8) |  | 9 (31.0) |  |
| *emergency c-section* |  | 33 (12.1) |  | 7 (10.3) |  | 4 (13.8) |  |
| *Complications during pregnancy* |  | 103 (37.9) |  | 26 (38.2) |  | 13 (44.8) | .764 |
| *Complications at birth* |  | 68 (25.0) |  | 24 (35.3) |  | 11 (37.9) | .109 |
| *Weight of child (g)* | 3410.7 (563.94) |  | 3432.87 (838.05) |  | 3472.07 (714.21) |  | .893 |
| *Subj. experience of traumatic childbirth* |  | 21 (7.7) |  | 12 (17.6) |  | 5 (17.2) | **.024** |
| *Relocation of child* |  | 68 (25.0) |  | 22 (32.4) |  | 10 (34.5) | .308 |
| *Breast feeding at childbirth* |  | 246 (90.8) |  | 66 (97.1) |  | 26 (89.7) | .216 |
| *Breast feeding at 12 weeks postpartum* |  | 226 (83.7) |  | 58 (85.3) |  | 22 (75.9) | .050 |
| *At least one stressful live event* |  | 129 (47.4) |  | 48 (70.6) |  | 22 (75.9) | **<.001** |
| *Number of stressful live events* | 0.86 (1.29) |  | 1.71 (1.77) |  | 1.9 (1.8) |  | **<.001** |
| *Number of childhood trauma* | 31.71 (9.13) |  | 36.57 (11.38) |  | 40.97 (13.98) |  | **<.001** |
| *emotional abuse* | 6.99 (3.07) |  | 8.65 (4.52) |  | 10.03 (4.83) |  | **<.001** |
| *physical abuse* | 5.49 (1.55) |  | 5.64 (1.47) |  | 6.52 (2.63) |  | **.004** |
| *sexual abuse* | 5.5 (1.89) |  | 5.86 (2.43) |  | 6.83 (3.61) |  | **.002** |
| *emotional neglect* | 7.59 (3.36) |  | 9.4 (4.13) |  | 10.24 (4.54) |  | **<.001** |
| *physical neglect* | 6.14 (1.99) |  | 7.03 (2.42) |  | 7.34 (3.02) |  | **<.001** |
| *PTSS score* | 5.21 (5.43) |  | 9.44 (7.35) |  | 14.03 (9.10) |  | **<.001** |
| *PMS severity* |  |  |  |  |  |  | **<.001** |
| *none* |  | 162 (59.6) |  | 23 (33.8) |  | 7 (24.1) |  |
| *mild* |  | 86 (31.6) |  | 26 (38.2) |  | 6 (20.7) |  |
| *severe* |  | 24 (8.8) |  | 19 (27.9) |  | 16 (55.2) |  |
| *MBQ score* | 8.2 (4.597) |  | 13.34 (4.625) |  | 15.79 (5.473) |  | **<.001** |
| *Baby blues* |  |  |  |  |  |  |  |
| *none* |  | 150 (55.1) |  | 12 (17.6) |  | 3 (10.3) | **<.001** |
| *moderate* |  | 113 (41.5) |  | 41 (60.3) |  | 13 (44.8) |  |
| *severe* |  | 9 (3.3) |  | 15 (22.1) |  | 13 (44.8) |  |
| *EPDS at childbirth* | 4.37 (3.02) |  | 10.75 (4.64) |  | 9.55 (4.23) |  | **<.001** |
| *EPDS 0-3 weeks pp* | 4.47 (2.91) |  | 9.85 (3.91) |  | 13.45 (6.12) |  | **<.001** |
| *EPDS 3-6 weeks pp* | 3.35 (2.80) |  | 8.43 (4.03) |  | 13.76 (4.35) |  | **<.001** |
| *EPDS 6-9 weeks pp* | 2.77 (2.72) |  | 7.19 (3.57) |  | 12.9 (4.23) |  | **<.001** |
| *EPDS 9-12 weeks pp* | 2.29 (2.47) |  | 5.63 (3.29) |  | 13.03 (3.52) |  | **<.001** |
| *MPAS 0-3 weeks pp* | 86.02 (5.64) |  | 82.01 (7.15) |  | 81.03 (8.06) |  | **<.001** |
| *MPAS 3-6 weeks pp* | 86.61 (5.38) |  | 82.99 (6.02) |  | 77.79 (10.93) |  | **<.001** |
| *MPAS 6-9 weeks pp* | 87.26 (4.95) |  | 84.28 (5.91) |  | 80.72 (9.12) |  | **<.001** |
| *MPAS 9-12 weeks pp* | 87.9 (4.7) |  | 85.18 (5.82) |  | 81.52 (7.91) |  | **<.001** |
| *PSS 0-3 weeks pp* | 13.21 (5.4) |  | 18.19 (5.36) |  | 22.76 (6.50) |  | **<.001** |
| *PSS 3-6 weeks pp* | 11.52 (4.99) |  | 17.15 (4.99) |  | 22.52 (6.56) |  | **<.001** |
| *PSS 6-9 weeks pp* | 10.43 (5.14) |  | 15.51 (5.86) |  | 21.31 (5.82) |  | **<.001** |
| *PSS 8-12 weeks pp* | 9.6 (5.27) |  | 13.35 (3.97) |  | 20.97 (4.54) |  | **<.001** |
| *Prior depression* |  | 27 (9.9) |  | 11 (30.9) |  | 15 (51.7) | **<.001** |
| *Familial psychiatric history* |  | 66 (24.3) |  | 25 (36.8) |  | 9 (31.0) | **.100** |
| *PBI: Care of own mother* | 29.96 (6.72) |  | 26.09 (8.02) |  | 26.56 (8.62) |  | **<.001** |
| *PBI: Overprotection of own mother* | 8.34 (6.42) |  | 10 (6.87) |  | 10.51 (7.24) |  | **.022** |
| *PBI Care of own father* | 27.33 (7.38) |  | 22.99 (8.70) |  | 20.8 (9.27) |  | **<.001** |
| *PBI: Overprotection of own father* | 7.31 (5.80) |  | 9.64 (7.22) |  | 11.79 (7.88) |  | **<.001** |
| *Support at home 0-12 weeks pp* | 1.73 (0.76) |  | 2.14 (1.12) |  | 2.55 (1.21) |  | **<.001** |

Note: PTSS: Premenstrual Tension Syndrome Scale; PMS: Premenstrual syndrome; MBQ: Maternity Blues Questionnaire; EPDS: Edinburgh Postnatal Depression Scale, MPAS: Maternal Postnatal Attachment Scale; PSS: Perceived Stress Scale; PBI: Parental Bonding Instrument; pp: postpartum

Table S2. Sample characteristics (means, standard deviations, and frequencies) of non-depressed postpartum women divided into women without baby blues and women with baby blues.

|  | No blues |  | Blues |  | p |
| --- | --- | --- | --- | --- | --- |
|  | **M (SD)** | **n(%)** | **M (SD)** | **n(%)** |  |
| *Age* | 33.24 (4.48) |  | 32.48 (3.95) |  | .07 |
| *Married* |  | 118 (78.7) |  | 93 (76.2) |  |
| *Education* |  |  |  |  |  |
| *< 9 years* |  | 9 (6.0) |  | 5 (4.1) |  |
| *10-12 years* |  | 28 (18.7) |  | 11 (9.0) |  |
| *> 13 years* |  | 113 (75.3) |  | 106 (86.9) |  |
| *Annual income* |  |  |  |  |  |
| *<20k €* |  | 15 (10.0) |  | 5 (4.1) |  |
| *20-50k €* |  | 40 (26.7) |  | 38 (31.1) |  |
| *>50k €* |  | 95 (63.3) |  | 80 (65.6) |  |
| *Primiparous* |  | 75 (50.0) |  | 80 (65.6) |  |
| *Total number of children* | 1.68 (0.79) |  | 1.48 (0.73) |  | **.016** |
| *Week of gestation* | 39.27 (1.52) |  | 39.06 (1.81) |  | .149 |
| *Weight of child (g)* | 3407.47 (524.42) |  | 3414.67 (611.22) |  | .459 |
| *Birth mode* |  |  |  |  | .181 |
| *spontaneous* |  | 84 (56.0) |  | 66 (54.1) |  |
| *Ventouse* |  | 9 (6.0) |  | 12 (9.8) |  |
| *elective c-section* |  | 34 (22.7) |  | 34 (27.9) |  |
| *emergency c-section* |  | 23 (15.3) |  | 10 (8.2) |  |
| *Complications during pregnancy* |  | 50 (33.3) |  | 53 (43.4) | .087 |
| *Complications at birth* |  | 39 (26.0) |  | 29 (23.8) | .673 |
| *Subj. experience of traumatic childbirth* |  | 10 (6.7) |  | 11 (9.0) |  |
| *Relocation of child* |  | 39 (26.0) |  | 29 (23.8) | .673 |
| *Breast feeding at childbirth* |  | 132 (88.6) |  | 114 (93.4) |  |
| *Breast feeding at 12 weeks postpartum* |  | 119 (80.4) |  | 107 (87.7) |  |
| *At least one stressful live event* |  | 72 (48.0) |  | 57 (46.7) |  |
| *Number of stressful live event* | 0.82 (1.13) |  | 0.91 (1.46) |  | .280 |
| *Number of childhood trauma* | 30.65 (7.54) |  | 33.01 (10.66) |  | **.017** |
| *emotional abuse* | 6.62 (2.56) |  | 7.45 (3.55) |  | **.014** |
| *physical abuse* | 5.41 (1.21) |  | 5.57 (1.90) |  | .201 |
| *sexual abuse* | 5.31 (1.11) |  | 5.74 (2.52) |  | **.032** |
| *emotional neglect* | 7.17 (3.07) |  | 8.11 (3.63) |  | **.012** |
| *physical neglect* | 6.13 (2.09) |  | 6.14 (1.86) |  | .481 |
| *PTSS score* | 3.77 (4.62) |  | 6.98 (5.83) |  | **<.001** |
| *PMS severity* |  |  |  |  | **<.001** |
| *none* |  | 110 (73.3) |  | 52 (42.6) |  |
| *mild* |  | 34 (22.7) |  | 52 (42.6) |  |
| *severe* |  | 6 (4) |  | 18 (14.8) |  |
| *EPDS at childbirth* | 3.99 (2.87) |  | 4.83 (3.13) |  | **.01** |
| *EPDS 0-3 weeks pp* | 2.77 (2.2) |  | 6.56 (2.23) |  | **<.001** |
| *EPDS 3-6 weeks pp* | 2.43 (2.48) |  | 4.47 (2.77) |  | **<.001** |
| *EPDS 6-9 weeks pp* | 1.95 (2.30) |  | 3.77 (2.86) |  | **<.001** |
| *EPDS 9-12 weeks pp* | 1.41 (1.88) |  | 3.38 (2.66) |  | **<.001** |
| *MPAS 0-3 weeks pp* | 87.73 (4.78) |  | 83.93 (5.9) |  | **<.001** |
| *MPAS 3-6 weeks pp* | 87.84 (4.94) |  | 85.11 (5.53) |  | **<.001** |
| *MPAS 6-9 weeks pp* | 88.36 (4.19) |  | 85.89 (5.47) |  | **<.001** |
| *MPAS 9-12 weeks pp* | 88.8 (4.42) |  | 86.8 (4.81) |  | **<.001** |
| *PSS 0-3 weeks pp* | 10.84 (4.61) |  | 16.13 (4.85) |  | **<.001** |
| *PSS 3-6 weeks pp* | 10.05 (4.73) |  | 13.32 (4.71) |  | **<.001** |
| *PSS 6-9 weeks pp* | 9.17 (5.03) |  | 11.98 (4.85) |  | **<.001** |
| *PSS 8-12 weeks pp* | 8 (4.75) |  | 11.56 (5.22) |  | **<.001** |
| *Prior depression* |  | 12 (8.0) |  | 15 (12.3) |  |
| *Familial psychiatric history* |  | 27 (18.0) |  | 39 (32.0) |  |
| *PBI: Care of own mother* | 30.07 (6.82) |  | 29.82 (6.62) |  | .379 |
| *PBI: Overprotection of own mother* | 7.45 (5.86) |  | 9.43 (6.92) |  | **.006** |
| *PBI Care of own father* | 28.36 (7.03) |  | 26.07 (7.63) |  | **.006** |
| *PBI: Overprotection of own father* | 6.7 (5.40) |  | 8.07 (6.19) |  | **.032** |
| *Support at home 0-12 weeks* | 1.69 (0.71) |  | 1.79 (0.82) |  | .137 |

Note: PTSS: Premenstrual Tension Syndrome Scale; PMS: Premenstrual syndrome; MBQ: Maternity Blues Questionnaire; EPDS: Edinburgh Postnatal Depression Scale, MPAS: Maternal Postnatal Attachment Scale; PSS: Perceived Stress Scale; PBI: Parental Bonding Instrument; pp: postpartum.

Table S3. Three-way contingency tables of frequency distributions between different combinations of postpartum diagnosis, baby blues severity, and PMS severity

|  |  | **no PMS** | **moderate PMS** | **severe PMS** |  |
| --- | --- | --- | --- | --- | --- |
| **No Blues** | ND | 110 | 34 | 6 | Fisher's exact = 18.68, *p* < .001 |
|  | AD | 4 | 3 | 5 |  |
|  | PPD | 1 | 2 | 0 |  |
| **Moderate Blues** | ND | 49 | 46 | 18 | Fisher's exact = 12.85, *p* = .01 |
|  | AD | 13 | 18 | 10 |  |
|  | PPD | 3 | 2 | 8 |  |
| **Severe Blues** | ND | 3 | 6 | 0 | Fisher's exact = 10.64, *p* = .025 |
|  | AD | 6 | 4 | 5 |  |
|  | PPD | 3 | 2 | 8 |  |
|  |  | ***no Blues*** | ***moderate Blues*** | ***severe Blues*** |  |
| **No PMS** | ND | 110 | 49 | 3 | Fisher's exact = 40.53, *p* < .001 |
|  | AD | 4 | 13 | 6 |  |
|  | PPD | 1 | 3 | 3 |  |
| **Moderate PMS** | ND | 34 | 46 | 6 | Fisher's exact = 11.29, *p* = .013 |
|  | AD | 3 | 18 | 4 |  |
|  | PPD | 2 | 2 | 2 |  |
| **Severe PMS** | ND | 6 | 18 | 0 | Fisher's exact = 18.27, *p* < .001 |
|  | AD | 5 | 10 | 5 |  |
|  | PPD | 0 | 8 | 8 |  |

Note: ND: Non-depressed; AD: Adjustment disorder; PPD: Postpartum depression; PMS: Premenstrual syndrome.
